# Inclusion of Fermented Cassava Top and Root with Inoculation in Total Mixed Ration Silage Diets: Ensilage Quality and Nutrient Digestibility of Backgrounding Crossbred Bulls

**DOI:** 10.3390/vetsci12050402

**Published:** 2025-04-24

**Authors:** Pichad Khejornsart, Unchan Traithilen, Theerayut Juntanam

**Affiliations:** Department of Agriculture and Resources, Faculty of Natural Resources and Agro-Industry, Kasetsart University, Chalermphrakiat Sakon Nakhon Province Campus, Sakon Nakhon 47000, Thailand; csnuct@ku.ac.th (U.T.); csntyj@ku.ac.th (T.J.)

**Keywords:** cassava top-root silage, crop–livestock production, TMR silage, rumen fermentation, beef cattle production

## Abstract

The use of cassava as a feed resource is the main focus of strategies that aim to increase the efficiency of beef cattle. In this study, the ratio of fresh cassava tops to roots (CTR) was 1:1, 1:2, 1:3, and 1:4 *w*/*w*, in addition to the incorporation of yeast and lactic acid bacteria (YL). CTRS was inoculated in vitro with rumen fluid and a substrate mixture to evaluate the gas production and fermentation quality. In vivo, crossbred beef cattle were randomly allocated to three dietary treatments: a control group and two groups that received total mixed ration (TMR) silage with either 35% or 70% of the concentrate replaced with CTRS. This study found that the levels of crude protein and condensed tannins (CTs) in CTRS were relatively increased by the addition of cassava tops (*p* < 0.05), while the fermentation pH decreased as the proportion of cassava roots increased. CTRS significantly enhanced in vitro dry matter digestibility, cumulative gas at 96 h, gas production kinetics, and MBP (*p* < 0.05), especially in the 1:2 group. In the in vivo experiment, CTRS increased DM intake, CP, and propionic acid concentration compared to the control group and showed great potential for improving NDF digestibility. Moreover, when the concentrate in the TMR silage was substituted with CTRS and offered to crossbred beef cattle, it increased ruminal NH_3_-N and blood glucose levels (*p* < 0.05). The TMR silage substituted with 70% CTRS reduced the feed cost by 50.54% (*p* < 0.05). Our study findings suggest that optimizing the inclusion of CTRS in TMR silage could be a viable strategy for beef producers aiming to enhance cattle performance and profitability.

## Simple Summary

Cassava (*Manihot esculenta*, Crantz) is commonly cultivated in subtropical and tropical regions and provides roots with a high soluble carbohydrate content but low crude protein content. Although the protein content of green top cassava leaves was high, it has been considered that incorporating cassava roots might increase the nutritional benefit of ruminant feed. This study explored the impact of adding cassava top-root silage (CTRS) to total mixed ration (TMR) silage on rumen fermentation and digestibility in beef cattle while lowering feed costs. This approach not only diversifies the feed options available to farmers but also helps reduce feed costs while maximizing the potential of locally sourced agricultural products.

## 1. Introduction

Recently, the limited amount of pasture available during the dry season has led to an increase in livestock production, which has been gradually increasing to feed confined cows with fresh-cut pasture daily; as a result, the need for enough feed is a tremendous challenge. Beef cattle play an important role in peri-urban agricultural production, which benefits sustainable agriculture [1,2], rural economies, and global food security [3], particularly in terms of animal protein food [4,5]. Thus, improving the sustainability of livestock production is critical, especially in terms of animal performance and economic efficiency. Even though the beef sector has been criticized for using significant amounts of grains to improve meat quality and growth performance, rising feed costs, especially of soybean meals and cassava chips, have resulted in lower farmer incomes. In order to compensate for the limitations in the quantity and quality of pasture or forage-based feed for the breeding herd and younger offspring before the finishing stage, strategic nutritional supplementation can be provided within these systems [2]. There is significant interest in investigating and developing alternative feeds that can replace concentrates and utilize local feed sources, particularly protein and energy resources. Crop–livestock production is of interest in terms of sustainability to protect the availability of ecosystems, especially in regard to growing competition for natural resources, decreasing land, climate change, and nutritional security [1].

Cassava (*Manihot esculenta*, Crantz), a starchy tuberous–crop, has been nutritionally evaluated as a feed for ruminant animals, especially in tropical and subtropical countries, due to its widespread availability, adaptability, and high carbohydrate content (81.0–87.1 g/100 g); furthermore, by lowering its protein levels (approximately 2.29–7.05%) [6,7,8], it can be used as a rapidly fermentable carbohydrate source [9]. Cassava is a major crop in Thailand, which ranks third in terms of economic significance behind rice and rubber. Cassava tops, a by-product of cassava cultivation, weigh 1441.5 kg/ha and are composed of leaves, petioles, and stems with 61.6%, 20.0%, and 18.4% dry matter (DM) [10]. Thus, Thailand’s cassava fields generate an estimated 2.25 million tons of green cassava top residue annually. A high protein content (20–30%) has been found in cassava tops and leaves [10,11,12]. It also has a high mineral content, including calcium (499.8 to 545.4 mg/100 g) and iron (129.1 to 146.1 mg/kg) [8]. However, it contains anti-nutritional components that need to be processed to reduce their toxicity, such as cyanogenic glucosides [12]. Anti-nutritional elements in leaves can be minimized by using the best processing techniques (such as boiling, fermentation, and sun-drying) [13]. Previous reports have shown that the cassava plant can be more successfully used in cattle diets when utilized fresh [14] and as dry chips, fermented roots with additives [15], pulp [7,16], and dried or ensiled leaves [17,18]. Even when cassava is used as animal feed, its roots are a rich source of energy, and its tops may be utilized as a source of protein for ruminant feed. However, during the rainy season, there is an insufficient supply of cassava chips to feed the cattle; thus, they become more costly. Furthermore, the low price of cassava roots and inconsistent marketing approaches have rendered cassava cultivation unprofitable. As such, both cassava tops and roots can be used to reduce feed costs and substitute the concentrates.

When farmers use ensilage technology, feed efficiency increases and expenses decrease. To meet the rising feed demand of high-yielding animals, a new feeding strategy has evolved beyond animal-based diets toward more readily fermentable feeds for ruminants [17,19,20]. These techniques promote the use of complete mixed ration ensiling for materials with excessive moisture. The potential for effectively managing ruminant diets is another advantage of feeding TMR silage compared to total mixed ration (TMR) [7]. Additionally, in recent years, the use of inoculation additives has been encouraged, and they have been shown to extend feed preservation by preventing spoilage, promoting progressive nutrient utilization, reducing anti-nutritional substances in feed, and improving fermentation quality, aerostability and digestibility of silage [15,16]. Nevertheless, challenges exist in using cassava as a substitute feed source in ruminant diets. Investigating efficient methods for fiber breakdown, additive fermentation, anti-nutrient reduction, and dietary integration is thus necessary. Therefore, this study’s goal was to assess the effects of backgrounding crossbred beef cattle on feed intake, digestibility, and rumen fermentation when CTRS was used instead of TMR silage concentrate.

## 2. Materials and Methods

### 2.1. Ethical Procedure

The research was conducted at the Animal Science Research Unit of the Faculty of Natural Resources and Agro-Industry, Kasetsart University, Chalermphrakiat Sakon Nakhon Province Campus, Sakon Nakhon, Thailand. All animal care and experimental procedures were approved by the Animals Ethical Committee of Kasetsart University (approval No. ACKU 60-ETC-004).

### 2.2. Preparation and In Vitro Assessment of TMR Silage

The cassava top-root silage (CTRS) was included as factor A and consisted of two different types of microbe inoculations: without microorganisms (M0) and yeast plus lactic acid bacteria (YL), and factor B was the ratio of fresh cassava leaves (CL) to fresh cassava roots (CR), which was 1:1, 1:2, 1:3, and 1:4 of *w*/*w* in this study. Microbial cultures were prepared according to the method described by the Thailand Institute of Science and Technology Research (TISTR). In brief, the production processes for the CTRS diet included *Saccharomyces cerevisiae* and *Lactobacillus casei* TISTR1463, which were cultivated separately in nutrient broth. Each culture was diluted with 0.05 M phosphate buffer (pH 7.0) until there were 108 cells/mL of microorganisms before the mixture was prepared. Fresh cassava roots and leaves (KU-72 varieties) were obtained from the Extension Farm Research Unit of Kasetsart University, Chalermphrakiat Sakon Nakhon Province Campus. They were harvested simultaneously from the roots and the leaves throughout the 240-day June cultivation cycle. To prepare CTRS, green top cassava, which was trimmed 30 to 50 cm from the apex and much above the root, was utilized. CTRS was mixed with varying proportions of chopped roots and leaves, supplemented with 15.17% rice bran and 1.5% molasses, and inoculated with 1% (10^8^ cells/mL) of the culture mixed with microbes (1:1 *v*/*v*), as shown in Table 1. This mixture was then placed in 150-L plastic barrels with tight lids and kept at 25 °C for 14 days. Silage samples were collected and analyzed for fermentation quality, including pH, organic acid content, and chemical composition.

The procedure of in vitro fermentation experiment was performed according to the procedure described by Menke and Steingass [21]. Briefly, 500 mg of substrate, 10 mL of rumen fluid, and 20 mL of artificial saliva, CO_2_ saturated, were added to a 60 mL vial. The TMR diet was used as a substrate for incubation and consisted of 48% rice straw, 12% cassava chips, 12% wet cassava pulp, 6.3% rice bran, 10% soybean meal, 6% palm kernel meal, 3.5% molasses, and 2.2% urea–mineral mix, and contained 43.2% NDF, 25.2% starch, and 10.6% CP on a DM basis. Three crossbred bulls were used as rumen fluid donors after being fed a TMR diet that included 43.2% NDF and 10.6% CP prior to morning feeding. Rumen fluid was collected via a stomach tube using a stainless–steel probe that was attached to an electric vacuum pump. After filtering the rumen contents through four layers of cheesecloth, the fluids were transported to the lab and combined with a medium for inoculation within 30 min in a CO_2_ atmosphere. Before sealing the bottles with butyl rubber stoppers and aluminum caps, the headspace was flushed again with CO_2_. Each treatment included six fermentation bottles, each containing three technical replicates and 4 bottles without blank substances. The bottles were incubated at 39 °C in a shaking incubator (WNB-29-Mammert, Schwabach, Germany) at 115 rpm for 96 h. Gas volumes were measured using the calibrated scale present on the syringes at 2, 4, 6, 8, 12, 16, 24, 32, 48, 72, and 96 h. Cumulative gas data were fitted using Ørskov and McDonald’s model [22].

### 2.3. Animals Trial

The CTRS with a 1:2 ratio of tops to leaves plus inoculation was selected for an animal trial to assess gas kinetics and IVDMD. Six healthy, one-year-old crossbred bulls weighing 175 ± 5.8 kg were randomly allocated to receive three different diets, which included two levels of CTRS replacement concentrates at 35 or 70% of TMR silage and a control treatment. This study used a double 3 × 3 Latin square design with three periods and treatments. The animals had access to mineral blocks and clean water in their individual confinements (2 m × 2.5 m × 1.5 m). The TMR silage was calculated based on the ingredients required by growing beef cattle [23], and the cattle were fed ad libitum in two equal meals at 08:30 h and 16:30 h daily to allow 10% refusals. The 63-day trial was divided into 21-day periods, with the remaining 7 days dedicated to sample collection and the remaining 14 days to treatment adaptation and feed intake measurements. A seven-day transition period was followed between the trials. Table 2 shows the chemical composition and feed components of the TMR silage, which included the CTRS used in the animal trials.

### 2.4. Sampling and Analytical Proceducres

#### 2.4.1. In Vitro Gas Production and Kinetics

The fermentation bottles were placed on ice to stop microbial activity after 24 h of incubation. After opening the bottles, a portable pH meter was used to determine the pH (HANNA Instruments HI 8424, Singapore). A 10 mL sample of fermentation liquid was mixed with 2 mL of 1M H_2_SO_4,_ and a 5 mL sample of fermentation liquid was stored at −20 °C for subsequent analysis of VFA and NH_3_-N, respectively. A 1-mL aliquot of the culture medium was collected in Eppendorf tubes and centrifuged at 16,000× *g* at 4 °C for 15 min to determine the concentrations of VFA via high-performance liquid chromatography (Agilent 1200 series, Agilent Technologies Inc. Santa Clara, CA, USA) with a diode array detector (Zorbax Eclipse XDB-C18 column (4.6 × 150 mm, 5 μm), Agilent Technologies Inc. Santa Clara, CA, USA) using 0.1 M phosphate buffer as the mobile phase), and ammonia nitrogen (NH_3_-N) using a micro Kjeldahl method (Distillation Unit K-355, BUCHI (Thailand) Ltd., Bangkok, Thailand), with only distillation and titration steps. Following fermentation, the nylon bags were washed with tap water and dried at 60 °C for 48 h to determine the in vitro dry matter digestibility (IVDMD). The Goering and Van Soest [24] methodology was used to calculate the true organic matter digestibility (TOMD), and Blummel and Lebzien’s [25] equation was used to estimate the microbial biomass production (MBP) of the TMR. Evaluations of ME were estimated using the GP value obtained after 24 h, and nutrients were analyzed using the equations by Menke and Steingass [21].

TOMD% = (OM initiation − OM residue)/(OM initiation) × 100(1)

MBP (mg) = TDOM (mg) − (Corrected gas production for 24 h × 2.20)(2)

where 2.20 is the stoichiometric factor for the mixed diets.

ME (MJ/kg DM) = 1.24 + 0.1457 GP24 + 0.0070 CP + 0.0224 CF(3)

where GP_24_ is the GP within 24 h of incubation (mL/500 mg DM), CP is the crude protein (g/kg DM), CA is the crude ash content (g/kg DM), and CF is the crude fat content (g/kg DM).

CTRS used as a substrate was carefully mixed, and representative samples were collected and analyzed for chemical composition and fermentation quality. Fermentative pH, ammonia-N, lactic acid, and volatile fatty acids (VFAs) were assessed as described by Franco et al. [26]. CTRS samples were dried at 60 °C for 48 h to a constant weight before processing them in a Cyclotech mill (Polymix^®^ PX-MFC 90D, Kinematica, Luzern, Switzerland) through a 1 mm screen. Dry matter (DM) was measured using gravimetry, ash content using muffle furnace incineration, ether extract (EE) using Soxhlet extraction, and crude protein (CP) using the Kjeldahl method from AOAC [27]. Neutral detergent fiber (NDF) and acid detergent fiber (ADF) were measured using the Van Soest method [28] with a fiber analyzer adapted to an Ankom Fiber Analyzer A2000 (Ankom Technology Corp., Fairport, NY, USA). The ether extract content was determined using the Soxhlet method. Condensed tannins (CTs) of the CTRS were estimated using the spectrophotometric HCl–butanol method described by Terrill et al. [29]. The non-fibrous carbohydrate (NFC) content was determined using the following equation: NFC = 100 − (%CP + %NDF + %EE + %ash).

#### 2.4.2. Animal Biological Response Evaluation

TMR silage dietary feed intake was assessed individually, and refusals were also recorded. Before the morning feeding time at the end of each period, the body weights of the bulls were measured. In the last five days of each period, various pens were used to examine the cattle’s digestibility. Fecal samples were collected rectally over a 5-day period when the cows were restrained in the stalls, with 2.5% subsamples stored at −20 °C before bulking within bulls and periods. The fecal samples used for N measurement were treated with 10 mL of 10% sulfuric acid to prevent ammonia volatilization. The samples (feed, refusals, and feces) were dried at 60 °C and ground (1 mm screen using Polymix^®^ PX-MFC 90D; Kinematica, Switzerland). The amounts of DM, ash, EE, CP [27], neutral detergent fiber (NDF), and acid detergent fiber (ADF) were assessed [28]. Acid-insoluble ash (AIA) was measured to determine the digestibility of nutrients [30].

In order to prevent saliva contamination, the earliest rumen fluid obtained was filtered. Rumen fluid was collected via oral tubes before feeding in the morning (6:00 h). The pH of the rumen fluid was immediately determined using a portable pH meter(HANNA Instruments HI 8424, Singapore). To determine the VFA concentration, 9 mL of rumen fluid was combined with 1 mL of 1M H_2_SO_4_ and centrifuged at 4 °C for 15 min at 16,000× *g* (Eppendorf centrifuged-5424R, Eppendorf Himac Technologies Co., Ltd., Takeda, Hitachinaka, Ibaraki, Japan), and the supernatant was frozen at −20 °C. VFA concentrations were determined using high-performance liquid chromatography (Agilent 1200 series, Agilent Technologies Inc. Santa Clara, CA, USA) with a diode array detector (Zorbax Eclipse XDB-C18 column (4.6 × 150 mm, 5 μm)) using 0.1 M phosphate buffer as the mobile phase, and ammonia nitrogen (NH_3_-N) using a micro-Kjeldahl method (Distillation Unit K-355, BUCHI (Thailand) Ltd., Bangkok, Thailand), with only distillation and titration steps. A vacuum blood collection tube was used to collect approximately 10 mL of blood from the jugular vein. Glucose, total protein, blood urea nitrogen (BUN), hemoglobin, and hematocrit levels were measured.

### 2.5. Statistical Analyses

All data from the in vitro experiment were statistically examined using SAS’s GLM procedure (Version 9.0; SAS Institute Inc., Cary, NC, USA), which employed a completely randomized design (CRD) with 2 × 4 factorial treatment arrangements. The following model was used to analyze the data:

Y_ijk_ = μ + α_i_ + β_j_ + αβ_ij_ + ε_ijk_ (4)

where Y_ijk_ = Observation values; μ = overall mean; α_i_ = effect of main factor A (microbial inoculation at i when i = M0 and YL); β_j_ = effect of main factor B (ratio of cassava tops to roots at j when j = 1:1, 1:2, 1:3, and 1:4); αβ_ij_ = interaction of A and B at ij; and ε_ijk_ is the residual error. At a significance level of *p* < 0.05, which is recognized as a statistically significant difference, the means were compared using Duncan’s new multiple-range tests.

The general linear model (GLM) in SAS software was used to examine the variances of the data using a 3 × 3 Latin square design. The model Y_ijk_ = μ + C_i_ + A_j_ + P_k_ + ε_ijk_ was used to evaluate the data, where Y_ijk_ is the observation from treatment i, cattle j, and period k; μ is the overall mean; C_i_ is the mean effect of the CTRS levels (i = 1–3); A_j_ is the mean effect of the animal (j = 1–3); P_k_ is the mean effect of the periods (k = 1–3); and ε_ijk_ is the residual error. To assess between-group differences in feed utilization, rumen fermentation parameters, and blood parameters of backgrounding crossbred bulls fed different diets with CTRS levels. The normality of the distribution of the variables was assessed using the Shapiro-Wilk test, which showed that all variables did not significantly deviate from the normal distribution (*p* > 0.05), and the assumption of normality was satisfied. Subsequently, the differences in treatment means were compared using Duncan’s new multiple-range tests. Using orthogonal polynomial contrasts, trends in treatments were statistically compared. A significance level of *p* < 0.05 was applied to determine whether the effect was substantial.

## 3. Results

### 3.1. Chemical Composition, Fermentation Quality, and In Vitro Trial

The proximate analysis of CTRS is presented in Table 3, which indicates that significant differences were found in the CP, DM, and fiber content of silage, and it did not assess the interaction between the ratio and inoculation. The inclusion of yeast-lactic acid bacteria (YL) in various ratios of cassava top-root silage, including a portion of cassava root, significantly reduced the total crude protein content and CT while increasing the NFC content (*p* < 0.05). Fermentative quality, in terms of pH and NH_3_-N concentration, significantly decreased (*p* < 0.05) with an increase in the level of cassava roots in CTRS silage, whereas the lactic acid content increased linearly (*p* = 0.012). The variables of fermentation quality, such as pH, NH_3_-N, and lactic acid, showed differences among treatments (*p* < 0.05), while acetic acid, propionic acid, and butyric acid concentrations were not different among treatments (*p* > 0.05). Inclusions with high levels of cassava roots and YL resulted in significantly reduced pH (*p* < 0.01) and NH_3_-N concentration (*p* < 0.01), whereas an increase in lactic acid concentration was observed (*p* < 0.01).

The data in Table 4 show the in vitro gas production kinetics and dry matter digestibility of CTRS with differences in the top-to-root ratio; the potential extent of gas production (a + b) and total gas production at 96 h showed significant interactions between the microbial (M) effect and the cassava top-to-root ratio (R) (*p* < 0.05). The cassava top-root ratio of 1:2 showed the lowest gas production from the immediately soluble fraction (a), the lowest gas production from the insoluble fraction (b), and the lowest gas potential extent of gas production (a + b) (*p* < 0.05). However, there were no differences between the treatment combinations for the gas production rate constant of the insoluble fraction (c). For the total gas accumulation at 96 h of incubation, the IVDMD and ME were different among the treatments (*p* < 0.05), but there was no difference in the inoculation effect (M) (*p* > 0.05). Microbial biomass production (MBP) did not interact with the main effect, although it had an impact when YL was not added and at a maximum ratio of 1:2 of CTRS (*p* < 0.05). The current results indicated that the effect of YL inoculation on total gas production was 98.48 and 94.06 mL/0.5 g higher than that of M0 at 96 h (*p* < 0.05), but there was no effect on the IVDMD and ME (*p* > 0.05). The cassava top-to-root ratio of 1:2 showed −2.98, 104.38, 101.41, and 99.49 mL/g and 62.42%, i.e., the highest a, b, a + b, and total gas production and IVDMD, respectively, compared to other treatments (*p* < 0.05). The MBP levels were between 112.25 and 127.17 mg. The top-to-root cassava ratio of 1:2 showed the best results, although YL to CTR inoculation did not affect MBP (*p* > 0.05).

### 3.2. Feed Intake and Nutrient Digestibility

The TMR silage diet was formulated by including the available high-moisture by-product, such as fresh cassava pulp; thus, TMR silage contained 43.23–45.26% DM (Table 5). The chemical composition of DM and the ADF content were low, while OM, EE, and NDF were unaffected. In TMR silage, the content of NFC and CT increased considerably in response to an increase in CTRS (*p* < 0.05). In contrast, when TMR silage was substituted with 70% CTRS, the feed cost was reduced by 50.54% (*p* < 0.05).

Table 6 shows the effect of CTRS as a substitute for concentrate in the TMR silage diets on feed intake and digestibility of nutrients in beef cattle. TMR silage included high levels of CTRS as the replacement of concentrate had significantly increased the feed intake and CT intake (*p* > 0.05), except for the intake of EE (*p* < 0.01). The group of 70% CTRS replacing concentrate showed a decline in CP (*p* = 0.080) and the NDF (*p* = 0.058) digestibility, although DM, OM, EE, and ADF were unchanged.

### 3.3. Ruminal Fermentation Characteristics, Blood Chemistry, and Hematology

The total volatile fatty acid, butyrate (C4), ruminal pH, and NH_3_-N were similar among the treatments (*p* > 0.05) after the consumption of TMR silage (Table 7). Propionate (C3) increased linearly when the concentrate in TMR silage was substituted with CTRS (*p* < 0.05); thus, the acetate-to-propionate ratio decreased (*p* = 0.080). The inclusion of CTRS as a 35–70% replacement of the concentrate in the TMR silage significantly increased the blood glucose concentration (*p* < 0.05) without adversely affecting the total protein, BUN, hemoglobin, and hematocrit levels (*p* > 0.05).

## 4. Discussion

### 4.1. Chemical Composition, Fermentation Quality, and In Vitro Trial

It is essential to optimize fermentation conditions, such as maintaining the temperature between 30 and 37 °C and lowering the pH below 4.5, in order to prevent infections and spoilage by bacteria. Safety and stability are further enhanced by fermenting in sterile, sealed containers and adding natural antimicrobials like salt or spices [31]. According to Duniere et al. [32], the lactobacilli population was not affected by *Saccharomyces* inoculation, but the number of CFUs increased with aerobic exposure to the silage, and this number was significantly higher than that of the terminal silage. However, a recent study suggested that a real fourth-generation inoculant may be produced by combining yeast with *Lactobacillus* species that promote ensiling, aerobic stability, and fiber digestion. Cassava tops have the potential to be utilized as a source of protein in cattle feed since they contain 23.0% crude protein (CP) [10]. Similar to Devi and Diarra’s [33] study, cassava leaf meal (CLM) has 167–399 g/kg of crude protein, 48–290 g/kg of crude fiber, 36–105 g/kg of ether extract, 57–125 g/kg of ash content, 314.7–450 g/kg of neutral detergent fiber and 6.7–10.2 MJ/kg of metabolic energy per kilogram of CLM. The CP of the CTRSsincreased when a high proportion of cassava leaves was incorporated (1:1 ratio), with or without YL inoculation (15.62 vs. 15.48%, respectively), although CT levels remained high (Table 3). Additionally, it may help improve the activity of rumen microorganisms and the digestion of nutrients. Cassava roots are an efficient energy source; however, their low protein content necessitates supplementation with other protein sources for optimal ruminant nutrition [6,9]. This highlights the importance of integrating both roots and leaves into cattle diets to achieve a balanced nutritional profile. Even toxification methods, such as fermentation and sun-drying, can enhance the nutritional value of cattle diets [8] and improve the growth performance of livestock when properly treated, as seen in studies with goats [34]. This study found that when CTRS was prepared with a 1:1 ratio of cassava tops to roots, its protein content increased by 15.62%. Gunan et al. [18] also found that CP was 13.6% when the cassava tops and roots were used in a 60:40 ratio. However, according to a study by Amos et al. [35], a 70:30 ensiled cassava root-to-top ratio had a CP of 10.1% and might potentially replace maize in the swine diet. The variable CP in CTRS may be due to variations in N fertilizer, harvest stage, soil, and cultivation. This might be an additional benefit, as the crude protein (CP) of CTRS can be used as a modifying substrate, resulting in optimal dietary CP concentrations for tropical beef cattle. The amount of NFC in the CTRS increases as the proportion of cassava root increases. This may be due to the high quantity of soluble carbohydrates and starch, which, in turn, results in a decline in fiber levels. Moreover, ensiling with YL inoculation resulted in a high lactic acid concentration. Flibert et al. [36] demonstrated that yeasts and LAB are associated with cassava fermentation and that the fermentation process contributes to a significant decrease in the cyanogen concentration of fresh cassava roots. Similarly, Damayanti et al. [37] demonstrated that *C. tropicalis* and *P. pentosaceus* can be employed as an inoculum in the cassava fermentation process to lower hydrogen cyanide (HCN) levels and increase cassava quality as a functional food. In comparison to the results obtained by Wanapat et al. [10], who showed that 4% of CT is present in cassava hay, Gunun et al. [18] observed that CARTOS with a top-to-root ratio of 60:40 contained 5.4% CT. This result is similar to that of the present study, in which a high ratio of cassava leaves (5.48%) from a 1:1 ratio of CTRS was found to possess a high CT concentration. According to Ragid et al. [38], cassava leaves contain a significant number of useful bioactive components, with biflavonoid isoginkgetin accounting for 25.33%. The primary phenolic components of the ethanolic extract of cassava leaves are rutin, gallic acid, and ferulic acid. This probably has an influence on the microbial activity during ensilage, as the NH_3_-N levels drop, corresponding to the increase in CT concentration in CTRS. This result is consistent with the findings of Jayanegara et al. [39], who utilized a meta-analysis to show that higher tannin concentrations were associated with lower quantities of soluble N, free amino acid N, non-protein nitrogen, and NH_3_-N in total silage N. The incorporation of energy and nitrogen in the best possible synchronization, which leads to ATP and N supply to microbial growth, is one way to improve ruminal microbial fermentation efficiency. The increase in in vitro DM digestibility observed in this study may be attributed to the stimulation of rumen microbial growth, which resulted in higher total gas production and better gas kinetic characteristics [2,40]. The mechanism underlying these beneficial effects has been attributed to improvements in short-chain fatty acid levels and microbial protein (MCP) formation by better synchronizing energy and N supply to the rumen. The ensiling combinations of cereal grains or grasses that are high in soluble carbohydrates and alfalfa in the right proportion have been shown to produce high-quality silages in certain experiments [41]. In this study, CTRS with a ratio of 1:2 showed that the quality of silage with 44.2% DM and 12.64% CP resulted in higher gas production (98.48 mL/0.5 g DM) and IVDMD (62.42) compared to other rations. According to the investigations by Badouei Dalfardi et al. [42], TMRS with 40% DM and 14.5% CP was a good way to preserve alfalfa hay since it had more DM, OM, and sensory evaluation in terms of quality and was in a better condition than other diets. Moreover, the higher IVDMD registered to increase the ratio of green top-root cassava could be due to the higher NFC content, as reported by Calabrò et al. [43].

### 4.2. Feed Intake and Nutrient Digestibility

The increased nutritional digestibility observed in this study may be attributed to the promotion of rumen microbial development, which resulted in increased feed degradability. Balanced nutrition may improve rumen microbe proliferation, resulting in enhanced rumen fermentation and fiber digestion [44]. The intake potential and energy availability of fodder crops in beef and dairy production are limited by their cell wall concentration and digestibility [45]. The cattle effectively consumed the TMR, which contained CTRS as a substitute for the concentrate. This was probably because of the reduction in rice straw proportion and an increase in the components of CTRS that were palatable, which were fermented with the YL inoculation to improve palatability. Providing more rumen-fermentable carbohydrates can be an effective feeding strategy, along with the provision of cassava top silage feed [6,10]. Moreover, administering cassava root silage supplements to grazing dairy cows improved their nutrient utilization and milk production, as reported by Galvão et al. [46]. The results of this study showed that while adding CTRS to the TMR silage diet had no effect on the consumption of other nutrients, it had a significant effect on the total dry matter intake (DMI).

The inclusion of CTRS in TMR silage did not significantly influence nutritional digestibility. When tannin-containing plants are added to animal diets, voluntary feed intake, and nutrient digestibility are often decreased. Despite improved ruminal fermentation and nitrogen utilization efficiency, our previous findings show that feeding beef cattle high tannin and polyphenol-containing plants, such as *Anacardium occidentale* L. and *Careya arborea* Roxb. leaf, can reduce their protozoal populations and nitrogen excretion without affecting their feed utilization or microbial nitrogen supply [2,47]. The occurrence of tannin–protein complexes increases when dietary tannins are included; thus, the bypass of proteins may be the cause of the lower proportion of CP in this study. Although Beauchemin et al. [48] reported that adding CTs at a concentration of more than 50 g/kg DM lowered the intake of DM or nutrients in goats, Avila et al. [49] showed that adding CTs at a concentration of more than 30 g/kg DM decreased the DMI of steers. However, we did not observe the aforementioned effect, most likely because the total diet had a lower CT content (11 and 20 g/kg DM for the 30 and 70% CTRS treatments, respectively). The results of this study showed significantly enhanced fiber digestion in cattle. However, tannins have an impact on protein and carbohydrate levels, especially cellulose, hemicellulose, pectin, and starch. Besharati et al. [50] demonstrated, using the most well-established methods, that tannins have long had a secondary anti-nutritional effect on the breakdown of fiber. These methods include the deprivation of nutrients, inhibitory effects on enzymes, direct action on rumen bacteria, and inhibition of rumen microbes’ enzymatic activity [51]. These results showed that YL addition supported rumen fermentation, and the bioactive compounds in CTRS may have contributed to providing a more favorable rumen environment, which may have encouraged a higher intake of fibrous materials. Furthermore, CTRS with YL may cause a loss of plant cell wall integrity during fermentation, and the subsequent fermentation of TMR silage by microorganisms may provide more soluble carbohydrates and, thus, a more acceptable odor. Animals that consume this kind of diet experience fermentation and the activity of ruminal microorganisms.

The primary sources of feed for ruminants are legumes and grasses, whereas, during the dry season, they continue to be fed low-quality roughage, particularly rice straw [4,10]. The efficiency of the estimated change in the optimized ranch income can be evaluated by analyzing the economic value of forage [52]. Additionally, ranchers and policymakers can prioritize conservation efforts to preserve or develop important forage resources by understanding the economic value of forage [53]. When CTRS was used as a substitute for concentrate in the TMR at 70%, it could efficiently reduce feed costs by over 50% compared with the control. In addition, the use of fermented feed is an effective and profitable economic strategy for feeding Hanwoo steers during the early and late fattening periods without causing adverse effects [54]. This investigation could be accomplished; however, the findings were limited to the evaluation of meat quality and animal performance. In order to increase the effectiveness of using the biomass that is already available and to lower the cost of feeding, it is imperative that low-cost alternative feed resources or new techniques and appropriate technology be developed.

### 4.3. Ruminal Fermentation Characteristics, Blood Chemistry and Hematology

The chemical composition of the feed material is a crucial component of the rumen fermentation process, as it affects the microbial community inside the ruminal consortium, especially through pH modulation. In this study, the ruminal pH dropped from 6.84 to 6.92 post-feeding, which is consistent with the observations of a previous study where high tannin and polyphenol-containing plant materials were added to the feed of beef cattle [47]. The pH of ruminal fluid frequently decreases in cattle fed high-concentrate and low-roughage diets; however, an increase in CTR levels had no detrimental effect on ruminal pH. A portion of the forage diet is substituted with an adequate proportion of readily fermentable carbohydrates to improve animal productivity and reduce rumen fiber degradation [9,55]. Furthermore, rumen fibrolytic fungi and the main fiber-degrading bacterial species, such as *Fibrobacter succinogenes*, *Ruminococcus albus*, and *R. flavefaciens,* are especially susceptible to rumen pH levels of 5.8 or below. Maximizing the balanced nutritional intake and availability, digestion, and, eventually, the efficiency of the milk and meat conversion process is the main focus of ruminant nutritionists. According to Gunun et al. [56], supplementing dairy cows with an MPM of 300 g/hd/d had no effect on their ruminal pH. Moreover, the rumen pH was within the optimal range of 6.5 to 7.0, demonstrating that the microbial species could break down the fiber in this pH range. Moreover, incorporating cassava pulp into TMR silage did not alter ruminal pH but effectively improved nutrient utilization in goats during their growth phase [7]. After feeding, the rumen NH_3_-N content increased due to the breakdown of ruminal dietary proteins. Most crude dietary proteins are broken down by bacteria in the rumen to NH_3_-N, which is the main source of nitrogen for rumen microbes, and a sufficient level of carbohydrates serves as a source of ATP [57]. Interestingly, the concentration of NH_3_-N tended to decrease when CTRS was added to the TMR silage. This may be associated with a decrease in protein digestibility, which may be impacted by CT. Notably, adding CTRS to TMR silage appeared to lower the concentration of NH_3_-N. This may be correlated with a decline in protein digestibility, which may be influenced by CT. Plant materials containing CT can bind to proteins to produce CT–protein complexes, which reduce the synthesis of NH_3_-N and the breakdown of proteins [54,58,59]. According to Koenig and Beauchemin [59], the impact of tannins on proteins is dependent on the number of hydrogen bonds that are stable between pH 3.5 and pH 8. Tannins may also release proteins in the abomasum if they dissociate from the proteins at pH 2.5 or 2.0. It is crucial to investigate ruminant metabolism, as the VFA serves as an energy source that supports their performance. One of the primary endproducts of carbohydrate metabolism in rumen microorganisms is acetic acid. The molar fraction of propionic acid increased linearly with the addition of the CTRS. Propionic acid may be produced in the rumen due to the optimal fermentation and breakdown of the proteins and carbohydrates by ruminal microbes present in CTRS, which may consequently increase the blood glucose levels of cattle. Similar to our results, cassava pulp fermented in traditional media of beef cattle showed an increase in the molar concentration of propionate [16]. However, Gunun et al. [18] observed that the replacement of concentrates with CARTOS reduced the nutrient intake, did not alter the blood glucose levels or the quantity of total VFA or propionic acid in the rumen, and resulted in a decline in the growth performance and feed utilization of beef cattle. The addition of CTRS had no effect on the hematologic state of cattle, which is commonly used to assess an animal’s nutrition and health. This may be because CARTOS has a larger proportion of cassava leaves than the other CTRS, and separate feeding is a different feeding practice. Although the addition of CTRS did not alter the total VFA levels, it increased the rumen’s propionic acid content, which may have contributed to improved growth performance. However, no inferences can be drawn about the growth performance of cattle, as our study did not thoroughly assess this parameter. BUN concentrations were correlated with NH_3_-N levels. Once ruminal NH_3_-N is absorbed into the bloodstream from the portal vein, the liver transforms it into urea. In this study, BUN levels were lower in cattle-fed CTRS diets. The BUN and hematocrit concentrations were not substantially impacted by the treatments (*p* > 0.05), indicating that the cattle’s general blood health and N balance were maintained [60]. Previous studies have shown that the observed BUN values, which range from 7 to 20 mg/dL, are normal. This indicates that the rumen has sufficient nitrogen for microorganisms to function adequately [61]. However, not all regions where cattle graze are suitable for growing protein-rich plants for their diets; especially tropical grasses have a low protein content. Although forages and concentrates with variable compositions and low nutrient quality occasionally fail to meet the ruminants’ nutritional needs, protein supplements, and other alternative nutritional sources are either expensive or unavailable. The findings of this study support sustainable beef production by utilizing agricultural by-products as important local feed resources through fermentation and a complete mixed ration feeding regime. However, a further and continual increase in beef cattle efficiency is required to fulfill the increasing human food demand while ensuring economic stability and environmental sustainability. Even though increasing the number of concentrates in beef cattle diets can enhance production efficiency, it reduces the benefits associated with ruminants’ capability to convert low-nutrient foods into high-quality milk and meat [3]. As nutrition has a significant influence on cattle efficiency, their dietary composition must be considered when assessing the environmental footprint of various livestock production methods. We also explored the challenges involved in effectively utilizing alternative feed supplies for cattle, such as nutrient composition changes, anti-nutritional factors, and the necessity for appropriate preprocessing and formulation strategies.

## 5. Conclusions

This study demonstrates the potential of utilizing cassava top-root silage, a high-value biomass, as an alternative feed for ruminants while assessing the positive impacts of this silage on in vitro rumen fermentation characteristics and microbial biomass production. Cassava top-root silage can be effectively incorporated into cattle diets, offering a sustainable and cost-effective alternative to traditional feeds. However, the level of inclusion of cassava-based silage in TMR silage is crucial; a moderate replacement (up to 70%) of the concentrate in the TMR silage with cassava-based silage maintains an adequate level of feed utilization in animals. Further research is required to evaluate the effects of cassava tops and roots incorporated in the TMR diet on the growth performance and meat quality of cattle, thereby providing deeper insights into the optimal feeding strategies and long-term impacts of condensed tannins on both animal productivity and environmental sustainability in tropical beef cattle production.

## Figures and Tables

**Table 1:** The ratio of cassava top-root silage (CTRS) was used in this experiment.

Inoculant	Ratio of Top-to-Root	Molasses, kg	Cassava Part		Rice Bran
			Green Top	Root	
M0	1:1	0.09	2.50	2.50	0.91
M0	1:2	0.09	1.67	3.33	0.91
M0	1:3	0.09	1.25	3.75	0.91
M0	1:4	0.09	1.0	4.0	0.91
YL	1:1	0.09	2.50	2.50	0.91
YL	1:2	0.09	1.67	3.33	0.91
YL	1:3	0.09	1.25	3.75	0.91
YL	1:4	0.09	1.00	4.00	0.91

M0 = without inoculation; YL = inoculation with yeast-lactic acid bacteria (*Saccharomyces cerevisiae* + *Lactobacillus casei (TISTR 1463)*).

**Table 2:** Ingredients and chemical composition of the TMR silage included the cassava top-root silage (CTRS) used in the animal experiments.

Item	Replacement of CTRS, %Concentrate		
	0	35	70
Ingredients, %dry matter			
Rice Straw	48.0	43.0	42.0
CTRS	0	20.3	40.0
Cassava Chip	12.00	8.23	3.80
Wet Cassava Pulp	12.00	8.23	3.80
Soybean meal	10.00	7.05	3.91
Rice bran	6.30	4.54	1.99
Palm kernel cake meal	6.00	4.33	1.90
Urea	0.70	0.60	0.57
Molasses	3.50	2.40	1.11
Sulfur	0.50	0.44	0.31
Salt	0.50	0.44	0.31
Mineral and vitamin mixture	0.50	0.44	0.31

**Table 3:** Chemical composition and silage quality of CTRS (*n* = 3).

Items	Inoculation (M)* and Ratio of Green Top-Root Cassava (R)													
	M0	M0	M0	M0	YL	YL	YL	YL	SEM	*p*-Value			Contrast	
	1:1	1:2	1:3	1:4	1:1	1:2	1:3	1:4		M	R	MR	L	Q
Chemical Composition, %DM														
DM	44.30	45.00	43.50	42.80	43.70	44.20	42.50	42.30	1.322	0.116	0.210	0.175	0.412	0.856
OM	91.40	90.70	90.70	91.10	90.80	90.82	90.90	91.40	1.524	0.155	0.087	0.552	0.472	0.172
CP	15.62 ^a^	12.84 ^ab^	11.45 ^bc^	10.68 ^c^	15.48 ^a^	12.64 ^ab^	11.23 ^bc^	10.49 ^c^	0.573	0.412	0.013	0.430	0.041	0.915
EE	2.17	2.14	2.11	2.10	2.38	2.35	2.26	2.17	0.150	0.159	0.501	0.140	0.285	0.142
NDF	42.41	40.95	40.84	39.51	42.86	41.63	39.60	39.15	1.095	0.173	0.185	0.343	0.172	0.423
ADF	27.31	25.91	25.15	25.15	26.16	25.27	24.50	26.67	0.714	0.611	0.235	0.911	0.681	0.107
NFC	30.19 ^b^	31.84 ^ab^	33.30 ^a^	33.32 ^a^	30.45 ^b^	32.90 ^b^	32.61 ^a^	32.96 ^b^	0.884	0.151	0.032	0.471	0.112	0.856
CT	5.48 ^a^	5.01 ^ab^	4.63 ^ab^	4.30 ^b^	5.45 ^a^	4.89 ^ab^	4.46 ^ab^	4.17 ^b^	0.335	0.814	0.021	0.170	0.093	0.451
Fermentation Quality, g/kg DM														
pH	5.12 ^a^	4.97 ^a^	4.50 ^b^	4.33 ^b^	4.31 ^b^	4.28 ^b^	4.25 ^b^	4.23 ^b^	0.129	0.010	0.024	0.035	0.014	0.131
NH_3_-N	2.62 ^a^	2.03 ^cd^	1.85 ^d^	1.76 ^d^	2.53 ^ab^	2.09 ^bc^	2.16 ^bc^	2.29 ^b^	0.108	0.050	0.072	0.086	0.713	0.170
Lactic acid	1.20 ^d^	1.32 ^d^	1.95 ^bc^	2.15 ^c^	3.09 ^b^	3.50 ^a^	3.49 ^a^	3.52 ^a^	0.099	0.010	0.034	0.418	0.012	0.147
Acetic acid	0.24	0.20	0.28	0.51	0.47	0.73	0.67	0.78	0.089	0.820	0.418	0.571	0.265	0.201
Propionic acid	0.010	0.012	0.017	0.023	0.025	0.030	0.029	0.026	0.008	0.258	0.126	0.295	0.226	0.215
Butyric acid	0.017	0.019	0.024	0.031	0.037	0.042	0.048	0.080	0.025	0.786	0.539	0.154	0.419	0.557

^abcd^ Means in the same row with different superscripts differ (*p* < 0.05). *M, Microbial inoculation; R, Ratio of green top-root cassava; DM, Dry matter; OM, Organic matter; CP, Crude protein; EE, Ether extract; NDF, Neutral detergent fiber; ADF, Acid detergent fiber; NFC, Non-fibrous carbohydrates; CT, Condensed tannin; NH_3_-N, Ammonia nitrogen; SEM, Standard error of the mean.

**Table 4:** In vitro gas production kinetics and digestibility of CTRS (*n* = 3).

Treatment		Gas Production Kinetics				Total Gas Accumulation 96 h, mL/0.5 g	IVDMD (%)	ME, MJ/kg DM	MBP, mg/g DM
M	R	a	b	c	a + b				
M0	1:1	−4.51 ^b^	99.77 ^b^	0.042	95.26 ^bc^	94.54 ^b^	60.05 ^b^	5.91 ^b^	123.47 ^a^
M0	1:2	−2.78 ^a^	101.87 ^ab^	0.044	99.09 ^ab^	97.67 ^ab^	61.96 ^ab^	6.06 ^b^	127.17 ^a^
M0	1:3	−3.71 ^ab^	100.70 ^ab^	0.052	96.99 ^b^	94.69 ^b^	57.74 ^bc^	5.97 ^b^	112.25 ^b^
M0	1:4	−3.01 ^a^	94.10 ^b^	0.050	91.09 ^c^	89.32 ^c^	56.68 ^c^	5.76 ^c^	118.09 ^ab^
YL	1:1	−4.37 ^b^	106.30 ^ab^	0.053	101.93 ^a^	99.70 ^a^	61.48 ^ab^	6.16 ^a^	121.00 ^ab^
YL	1:2	−3.17 ^a^	106.89 ^a^	0.045	103.72 ^a^	101.30 ^a^	62.88 ^a^	6.23 ^a^	125.01 ^a^
YL	1:3	−3.29 ^ab^	103.74 ^ab^	0.048	100.45 ^ab^	98.80 ^ab^	60.26 ^ab^	6.13 ^ab^	116.61 ^b^
YL	1:4	−3.01 ^a^	98.37 ^b^	0.049	95.36 ^bc^	94.14 ^b^	59.20 ^bc^	5.96 ^bc^	119.98 ^ab^
SEM		0.397	2.283	0.015	1.534	1.467	0.917	0.067	3.109
Factor M									
M0		−3.50	99.11	0.047	95.61 ^b^	94.06 ^b^	59.11	5.93	120.24
YL		−3.46	103.83	0.049	100.37 ^a^	98.48 ^a^	60.96	6.12	120.65
Factor R									
1:1		−4.44 ^b^	103.04 ^ab^	0.048	98.60 ^a^	97.12 ^a^	60.77 ^a^	6.04 ^ab^	122.24 ^ab^
1:2		−2.98 ^a^	104.38 ^a^	0.045	101.41 ^a^	99.49 ^a^	62.42 ^a^	6.15 ^a^	126.09 ^a^
1:3		−3.50 ^ab^	102.22 ^ab^	0.050	98.72 ^a^	96.74 ^a^	59.00 ^b^	6.05 ^ab^	114.43 ^b^
1:4		−3.01 ^a^	96.24 ^b^	0.050	93.23 ^b^	91.73 ^b^	57.94 ^b^	5.86 ^b^	119.03 ^ab^
Interaction M × R		0.612	0.771	0.251	0.034	0.048	0.642	0.327	0.108
Contrast									
L		0.067	0.050	0.114	0.050	0.050	0.024	0.030	0.117
Q		0.287	0.314	0.512	0.228	0.147	0.117	0.217	0.210

SEM, Standard error of the mean; M, Microbial inoculation (M0 = without inoculation; YL = inoculation with yeast-lactic acid bacteria); R, Ratio of green top-root cassava; L, Linear; Q, Quadratic; IVDMD, in vitro dry matter digestibility; MBP, microbial biomass production; a = gas production from the immediately soluble fraction; b = gas production from the insoluble fraction; c, the gas production rate constant for the insoluble fraction (b); a + b, the gas potential extent of gas production. ^abc^ Means in the same row with different superscripts differ (*p* < 0.05).

**Table 5:** The chemical composition of TMR silage with the inclusion of CTRS (*n* = 3).

Item	Cassava Top-Root Silage (CTRS), %Concentrate			SEM	*p*-Value	Contrast	
	0	35	70			L	Q
Chemical composition, % of DM							
DM	45.63	43.68	43.23	1.245	0.065	0.098	0.246
OM	87.85	88.50	87.78	2.478	0.571	0.128	0.306
CP	10.62	10.60	10.54	0.255	0.245	0.144	1.424
EE	2.57	2.94	3.12	0.197	0.115	0.099	0.123
NDF	58.48	56.84	54.85	1.542	0.478	0.064	0.153
ADF	38.48	36.84	34.85	0.927	0.054	0.072	0.713
NFC	20.18 ^b^	22.12 ^ab^	23.27 ^a^	1.025	0.041	0.010	0.219
CT	0.58 ^b^	1.02 ^ab^	2.00 ^a^	0.452	0.010	0.017	0.137
Price (USD/100 kg DM)	16.68 ^a^	12.82 ^ab^	8.25 ^b^	1.579	0.014	0.021	0.127

^ab^ Means in the same row with different superscripts differ (*p* < 0.05); DM, Dry matter; OM, Organic matter; CP, Crude protein; EE, Ether extract; NDF, Neutral detergent fiber; ADF, Acid detergent fiber; NFC, non-fibrous carbohydrates; CT, Condensed tannin; L, linear; Q, quadratic; SEM, Standard error of the mean.

**Table 6:** The effect of CTRS as a substitute for concentrate in TMR silage diets on feed intake and digestibility of nutrients in beef cattle.

Item	Cassava Top-Root Silage (CTRS), %Concentrate			SEM	*p*-Value	Contrast	
	0	35	70			L	Q
Dry matter intake							
kg/d	5.78 ^b^	5.98 ^ab^	6.03 ^a^	0.145	0.041	0.011	0.572
%BW	3.34	3.40	3.34	0.325	0.230	0.472	0.594
Nutrient intake, kg/d							
OM	5.08 ^b^	5.29 ^a^	5.29 ^a^	0.156	0.047	0.023	0.751
CP	0.61	0.63	0.64	0.086	0.065	0.102	0.515
EE	0.15	0.18	0.19	0.039	0.082	0.098	0.118
NDF	3.38	3.40	3.31	0.124	0.107	0.183	0.270
ADF	2.22	2.00	2.20	0.108	0.112	0.120	0.127
CT	0.03 ^b^	0.06 ^ab^	0.11 ^a^	0.020	0.010	0.051	0.214
Digestibility coefficients, %							
DM	59.87	61.95	60.80	1.090	0.069	0.104	0.170
OM	61.39	63.28	62.88	1.990	0.341	0.390	0.119
CP	64.79	63.90	62.65	1.458	0.080	0.690	0.430
EE	54.64	56.32	55.97	1.751	0.594	0.829	0.516
NDF	48.86	50.05	49.95	1.840	0.058	0.090	0.232
ADF	39.79	40.99	41.59	1.036	0.145	0.085	0.513

^ab^ Means in the same row with different superscripts differ (*p* < 0.05); DM, Dry matter; OM, Organic matter; CP, Crude protein; EE, Ether extract; NDF, Neutral detergent fiber; ADF, Acid detergent fiber; CT, Condensed tannin; L, linear; Q, quadratic; SEM, Standard error of the mean.

**Table 7:** The effect of CTRS as a substitute for concentrate in TMR silage diets on rumen fermentation characteristics, blood chemistry, and hematology of beef cattle.

Item	Cassava Top-Root Silage (CTRS), %Concentrate			SEM	*p*-Value	Contrast	
	0	35	70			L	Q
Rumen fermentation parameters							
pH	6.84	7.02	6.89	0.032	0.450	0.700	0.547
NH_3_-N (mg/dL)	18.5	17.4	16.7	1.036	0.071	0.099	0.772
Total volatile fatty acid (mM/L)	99.7	105.1	102.5	5.250	0.342	0.241	0.597
Volatile fatty acid (mol/100 mol)							
Acetate	65.2	64.2	64.5	1.066	0.218	0.131	0.945
Propionate	19.5 ^b^	21.3 ^a^	20.6 ^ab^	0.545	0.050	0.042	0.544
Butyrate	15.2	14.9	14.8	0.497	0.714	0.141	0.950
Acetate-to-propionate ratio	3.3	3.0	3.1	0.069	0.180	0.163	0.359
Blood chemistry and hematology							
Glucose (mg/dL)	76.93 ^b^	78.84 ^ab^	81.72 ^b^	1.296	0.048	0.012	0.348
Total protein (g/dL)	5.94	6.04	6.51	0.338	0.168	0.330	0.093
BUN (mg/dL)	8.70	8.02	7.78	0.485	0.060	0.315	0.220
Hemoglobin (g/dL)	5.84	7.86	7.61	0.788	0.211	0.670	0.090
Hematocrit (%)	17.53	20.08	19.93	0.894	0.324	0.680	0.141

^ab^ Means in the same row with different superscript differ (*p* < 0.05); BUN, blood urea nitrogen; L, linear; Q, quadratic; SEM, Standard error of the mean.

## Data Availability

The original contributions presented in this study are included in the article material. Further inquiries can be directed to the corresponding author.
